# European Association of Endoscopic Surgery Clinical Practice Guideline Protocol: Evidence‐Informed Recommendations on the Management of Cholecystitis With ESAIC and ESR Participation

**DOI:** 10.1002/gin2.70043

**Published:** 2025-09-01

**Authors:** Bright Huo, Salvador Morales Conde, Carolina Soledad, Alexander A. Tzanis, Alexandros‐Georgios Asimakopoulos, Dimitris Mavridis, Gordon Guyatt, Stavros A. Antoniou

**Affiliations:** ^1^ Department of Surgery, Division of General Surgery McMaster University Hamilton Canada; ^2^ Department of General and Digestive Surgery University Hospital Virgen Macarena, University of Seville Seville Spain; ^3^ Anesthesia and Critical Care Department University General Hospital Valencia Valencia Spain; ^4^ Research Methods Department Universidad Europea de Valencia Valencia Spain; ^5^ First Department of Obstetrics and Gynecology Alexandra University Hospital, National and Kapodistrian University of Athens Athens Greece; ^6^ Department of Primary Education, School of Education University of Ioannina Ioannina Greece; ^7^ Department of Health Research Methods, Evidence, and Impact, Department of Medicine McMaster University Hamilton Canada; ^8^ Department of General Surgery Papageorgiou General Hospital Thessaloniki Greece

**Keywords:** cholecystitis, EAES, percutaneous drainage, surgery

## Abstract

**Background:**

Several options exist for the management of acute cholecystitis. However, practice patterns vary widely, and there is a need for high‐quality guidance in areas of controversy.

**Methods and Analysis:**

We will develop a clinical practice guideline on the management of patients with Tokyo Grades I–III acute cholecystitis. The guideline will answer the following key questions:

1. Should adult patients with Grades I or II acute cholecystitis receive conservative management or emergent laparoscopic cholecystectomy?
2. Should adult patients with Grades II or III acute cholecystitis receive percutaneous drainage or emergent laparoscopic cholecystectomy?
3. Should adult patients with Grades II or III acute cholecystitis receive conservative management or emergent laparoscopic cholecystectomy > 72 h from symptom onset?

Our systematic review group will undertake multiple systematic reviews to identify articles addressing each key question. Next, we will appraise the certainty of the evidence using the Grading of Recommendations Assessment, Development, and Evaluation (GRADE) methodology. An international, expert multidisciplinary panel will then meet to review the evidence and develop recommendations using GRADE's evidence‐to‐decision framework. This panel will consist of four general surgeons, an interventional radiologist, an intensive care specialist, an anaesthetist, and two patient representatives. Panel members will finalize our recommendations by consensus at an in‐person meeting. This guideline will adhere to methodological standards according to GIN, GRADE and AGREE‐S.

**Ethics and Dissemination:**

All participating members will declare their conflicts of interest, which will be addressed prior to guideline development. This clinical practice guideline will be presented at international scientific meetings, online presentations and social media, and published in full in the journal of *Surgical Endoscopy & Other Interventional Techniques*.

## Summary

- Literature for the management of cholecystitis is mixed.
- Our updated evidence synthesis will address contemporary questions in the surgical management of cholecystitis.
- We will use these findings to develop evidence‐informed recommendations for patients with cholecystitis.

## 1 Introduction and Scope

Laparoscopic cholecystectomy is considered the standard of care in the management of patients with acute calculous cholecystitis [1]. However, surgical intervention for acute cholecystitis is not without complications. Laparoscopic cholecystectomy is associated with a 30‐day mortality rate as high as 1% for patients with Grades I (mild) or II (moderate) cholecystitis, defined by the Tokyo criteria [2]. Moreover, the reported conversion rate from laparoscopic to open surgery ranges from 2.4% to 25.6% in this population [2]. Further, postoperative complications range from 2.9% to 9.7% for Grade I cholecystitis and 3.1% to 28.0% for Grade II cholecystitis [2].

These issues raise the possible role of nonoperative management in select patients with acute cholecystitis. In patients with Grades I or II cholecystitis, recent literature suggests that the use of conservative management with antibiotics or percutaneous drainage is a viable alternative to laparoscopic cholecystectomy, particularly among the elderly [3]. In contrast, others cite an increased rate of major complications with percutaneous drainage compared to laparoscopic cholecystectomy [4].

In this context, there is a need for high‐quality, evidence‐informed guidance on the management of patients with acute cholecystitis that are unaddressed by existing guidelines [2]. This protocol outlines an initiative by the European Association of Endoscopic Surgery (EAES) and our robust methodological standards for the develop evidence‐informed recommendations to guide general, hepatobiliary, gastrointestinal and acute care surgeons, as well as primary care and internal medicine physicians and interventional radiologists involved in the management of patients with mild and moderate acute cholecystitis. Our objective is to minimize morbidity and optimize the safe and effective care of patients with acute cholecystitis.

## 2 Methods

### 2.1 Steering Group

The steering group will consist of three co‐chairs. These members include a supervising methodological chair (Stavros A. Antoniou) who is a certified lead guideline developer and chair (International Guideline Development Credentialing & Certification Program – INGUIDE Certificate #2022‐L3‐V1‐00014). This senior methodological co‐chair will supervise a junior methodological co‐chair (Bright Huo) who is a certified guideline methodologist (INGUIDE Certificate #2023‐L2‐V1‐00255). Finally, a surgeon (Salvador Morales Conde, S. M. C.) with content expertise in hepatobiliary surgery and prior participation in guideline development will act as the content expert co‐chair.

### 2.2 Guideline Panel

We will consider members with relevant clinical and research backgrounds. The selection of panel members will also ensure a balanced geographical representation of members from countries across Europe, as well as age and gender diversity.

The multidisciplinary, expert guideline panel will include four gastrointestinal surgeons, members of the EAES, with experience in treating patients with acute cholecystitis. The panel will further comprise one interventional radiologist, an intensive care specialist, an anaesthetist, and two patient partners. These ten panel members will be free of significant intellectual conflicts of interest and recognized as full voting members during the synchronous consensus meeting, including patient partners. One patient partner has had a previous laparoscopic cholecystectomy after prolonged presentations with recurring episodes of pain with an unremarkable postoperative course. The other patient partner has had experience in participating in previous guideline panels and has become a certified guideline panel member through the INGUIDE programme (INGUIDE Certificate #20250605‐C1‐V2‐SD‐2178). She will provide informal mentorship for the other patient partner with respect to the guideline panel discussion and conduct. Finally, the panel will be completed by one GRADE Auditor who is an original co‐author of GRADE, Gordon Guyatt. This member will ensure that our guideline development processes adhere to the core tenets of GRADE and will act as a non‐voting member. We will apply the International Committee of Medical Journal Editors criteria for authorship to consider any member of the panel for authorship in the peer‐reviewed publication of our final guideline report [5].

### 2.3 Systematic Review Group

This team will be comprised of systematic reviewers, statisticians, methodologists, and the GRADE advisor. The systematic review group will be led by the Systematic Review Lead (Alex Tzanis, A. T.) who will coordinate and oversee all activities of the group. Two trainees (Konstantinos Perivoliotis and Fani Apostolidou Kiouti) will be involved in this team and have completed a structured curriculum on systematic review methodology: https://drive.google.com/drive/folders/1hbw6fZIZU4idPcaq5y1buVfdLeXDLMIB.

A third trainee (Elisa Calabrese) who has completed the SAGES guideline curriculum will complete the systematic review group. An expert statistician (Dimitris Mavridis) with experience in data analysis for over 15 clinical practice guidelines will lead the statistical analysis. This senior lead analyst will guide a junior lead analyst (Alexandros‐Georgios Asimakopoulos) in performing all data analysis for the systematic review and resulting clinical practice guideline. The junior co‐chair will liaise between the systematic review group and the steering group as the methodological co‐chair.

### 2.4 Conflicts of Interest

All three members of the steering group have no personal, financial, or intellectual conflicts of interest. The steering group will collect conflicts of interest from all members involved in the development of this guideline and will adjudicate them according a previously described framework [6]. Conflicts of interest to be collected will include personal, intellectual, or financial conflicts. Panel members identified as having significant conflicts of interest will act as external advisors during the in‐person consensus meeting and will participate in all discussions related to the evidence synthesis review and evidenceto‐ decision framework but will not vote or participate in the discussion related to the development of the final recommendations. The GRADE auditor will ensure that this process is adjudicated appropriately. Detailed questions regarding conflicts of interest are summarized in Supporting Information S1: Appendix 1.

### 2.5 Guideline Development Process

This guideline protocol adheres to the best applicable reporting standards from the Preferred Reporting Items for Systematic reviews and Meta‐Analysis Protocols [7]. The guideline will follow rigorous methodological standards set by the Grading of Recommendations Assessment, Development, and Evaluation (GRADE) framework [8], and will address key points in the Appraisal of Guidelines for Research and Evaluation (AGREE) II extension for surgical interventions (AGREE‐S) [9].

We registered this guideline proposal on the International Guidelines Library, available here. EAES members will have the opportunity to provide feedback on this protocol through social media as well as via the EAES newsletter. The steering group will consider all suggestions and action revisions to the protocol as needed. We will then transparently report in the guideline manuscript any amendments made to the study design following the publication of this protocol, with justification.

### 2.6 Research Questions

This guideline topic was informed by an internal biennial survey of EAES members that vote on priority topics for future guidelines. Our steering group determined the following key questions:

1. Should adult patients with Grades I or II acute cholecystitis receive conservative management or laparoscopic cholecystectomy?
2. Should adult patients with Grades II or III acute cholecystitis receive percutaneous drainage or laparoscopic cholecystectomy?
3. Should adult patients with Grades II or III acute cholecystitis receive conservative management or laparoscopic cholecystectomy > 72 h from symptom onset?

The guideline questions will consider the institution's expertise in advanced laparoscopic surgery, the individual surgeon's experience, and the availability of human and material resources for performing either intervention. We will perform subgroup analyses for anaesthetic risk, frailty, Tokyo grade, and comorbidity status, as the data allows.

### 2.7 Systematic Review

The Systematic Review Lead will develop a systematic literature search under the supervision of an academic health sciences librarian who is an expert in systematic reviews. A. T. is an experienced systematic reviewer and will oversee the systematic review process by leading an evidence synthesis team.

We will query online databases including PubMed, Embase via Elsevier and Cochrane Central Register of Controlled Trials to identify eligible articles of interest published prior to March 14, 2025 (Supporting Information S2: Appendix 2). The team will use the inclusion and exclusion criteria listed in Tables 1, 2, 3 to select articles for inclusion.

**Table 1:** Inclusion and exclusion criteria for KQ #1.

KQ #1	Inclusion criteria	Exclusion criteria
Design	RCTs Comparative studies: ∘ Cohort studies ▪ Prospective ▪ Retrospective ∘ Case‐control studies ▪ Applying propensity‐score matching or logistic regression	Non‐primary literature ∘ Systematic reviews (SR), meta‐analyses (MA) ∘ Narrative reviews ∘ Standards of practice ∘ Commentaries ∘ Editorials ∘ Letters to the editor ∘ Opinion articles Case series Case reports Cross‐sectional studies Single‐arm studies
Population	Adult patients with Grades I or II acute cholecystitis	Adults with Grade III acute cholecystitis Animal studies Veterinary studies Paediatric studies Pregnancy
Intervention	Conservative management	
Comparators	Laparoscopic cholecystectomy	Percutaneous drainage
Aims	Studies assessing clinical outcomes between conservative management and laparoscopic cholecystectomy	Studies assessing basic science topics related to the management of cholecystitis

**Table 2:** Inclusion and exclusion criteria for KQ #2.

KQ #2	Inclusion criteria	Exclusion criteria
Design	RCTs Comparative studies: ∘ Cohort studies ▪ Prospective ▪ Retrospective ∘ Case‐control studies ▪ Applying propensity‐score matching or logistic regression	Non‐primary literature ∘ Systematic reviews (SR), meta‐analyses (MA) ∘ Narrative reviews ∘ Standards of practice ∘ Commentaries ∘ Editorials ∘ Letters to the editor ∘ Opinion articles Case series Case reports Cross‐sectional studies Single‐arm studies
Population	Adult patients with Grades II or III acute cholecystitis	Adults with Grade I acute cholecystitis Animal studies Veterinary studies Paediatric studies Pregnancy
Intervention	Percutaneous drainage	
Comparators	Laparoscopic cholecystectomy	Conservative management
Aims	Studies assessing clinical outcomes between percutaneous drainage and laparoscopic cholecystectomy	Studies assessing basic science topics related to the management of cholecystitis Studies assessing nonoperative management related to the management of cholecystitis

**Table 3:** Inclusion and exclusion criteria for KQ #3.

KQ #3	Inclusion criteria	Exclusion criteria
Design	RCTs Comparative studies: ∘ Cohort studies ▪ Prospective ▪ Retrospective ∘ Case‐control studies ▪ Applying propensity‐score matching or logistic regression	Non‐primary literature ∘ Systematic reviews (SR), meta‐analyses (MA) ∘ Narrative reviews ∘ Standards of practice ∘ Commentaries ∘ Editorials ∘ Letters to the editor ∘ Opinion articles Case series Case reports Cross‐sectional studies Single‐arm studies
Population	Adult patients with Grades II and III acute cholecystitis and symptom onset > 72 h	Adults with Grade I acute cholecystitis Animal studies Veterinary studies Paediatric studies Pregnancy
Intervention	Conservative management	
Comparators	Laparoscopic cholecystectomy	Conservative management
Aims	Studies assessing clinical outcomes between conservative management and laparoscopic cholecystectomy	Studies assessing basic science topics related to the management of cholecystitis Studies assessing nonoperative management related to the management of cholecystitis

We will upload articles from all database searches to Covidence. Next, A. T. will lead calibration for the first round of screening using a sample of ten abstracts. This will be done live with the two trainee reviewers, with iterative feedback. Once calibration is complete, the two trainee reviewers will complete the first round of screening. The same process will be repeated for the second round of screening. Similarly, A. T. will lead a calibration session prior to beginning data extraction using five articles. Both trainee reviewers will perform data extraction for all included articles. Throughout both rounds of screening and data extraction, A. T. will be available to resolve disagreements as they arise and will hold a synchronous meeting to resolve discrepancies if needed. We will extract the following variables into Covidence:

- Study number
- Author's last name
- Year of publication
- Title
- Country
- Study design
- Inclusion criteria
- Exclusion criteria
- Age (mean ± SD)
- ASA class
- APACHE II score
- Frailty
  ∘ For example: modified frailty index
- BMI (kg/m^2^)
- Prior abdominal surgery
- Number of episodes of cholecystitis
- Comorbidities
  ∘ Hypertension
  ∘ Diabetes mellitus
  ∘ COPD
  ∘ MI
    ▪ If reported, whether MI within 12 months
  ∘ Stroke
    ▪ If reported, whether MI within 12 months
- Surgical technique
- Number of patients with acute cholecystitis who underwent surgery in the institution where the study was conducted
- Expertise of centre in advanced laparoscopic procedures
- Experience of operating surgeons in advanced laparoscopic procedures
- Outcomes (to be prioritized by the panel)
  ∘ 90‐day mortality
  ∘ Length of stay
  ∘ Quality of life at 5 years
  ∘ Readmission at 90 days
  ∘ (Re)intervention (surgical, radiological, or endoscopic related to biliary disease) at 1 year
  ∘ Recurrent biliary disease at 1 year (cholecystitis + cholangitis + biliary colic)
  ∘ Recurrent biliary disease at 1 year (cholecystitis + cholangitis)
  ∘ 30‐day major complications (≥ 3b)
- Follow‐up for outcome(s) in months
- Comments/notes

The systematic review group will complete all stages of the systematic review process. They will perform two rounds of screening by title and abstract followed by full‐text, data extraction and risk of bias appraisal, overseen by the Systematic Review Lead as a third reviewer.

### 2.8 Outcome Prioritization

The junior co‐chair will draft a list of potential outcomes after conducting a scoping review of recently published studies. Preference will be given to articles published in the last two decades to ensure the contemporary relevance of our findings. The supervising co‐chair and the content expert co‐chair will also suggest outcomes as appropriate. The junior co‐chair will then aggregate a comprehensive list of potential outcomes for review by S. A. A. and S. M. These co‐chairs will revise these outcomes based on their clinical experience.

We will then present these outcomes to panel members, who will have the opportunity to propose additional outcomes for consideration. The panel will rate the importance of each outcome using the GRADE scale to identify important (Scores 4–6) and critical (Scores 7–9) items for inclusion [10]. If significant variation exists among panel member ratings, the steering group will hold a synchronous panel meeting to address disagreements and reach consensus. To guide this discussion, the group will follow GRADE guidance in focusing on patient‐important outcomes relevant for clinicians [10].

### 2.9 Minimal Important Differences

We will distribute an online survey to panel members to define minimal important differences using predefined approaches for guideline panel surveys [11]. In line with this framework, we will present panellists with the baseline risks and corresponding decrease in harmful outcomes or increase in positive outcomes, respectively. Questions will be framed as in the following theoretical example:

‘Laparoscopic cholecystectomy reduces the risk of recurrent admissions at 90 days by 50 in 1000 patients among those with Grades I or II acute cholecystitis.

What is the magnitude of effect?

- All or almost all would consider this an important effect
- Most would consider this an important effect
- A majority would consider this an important effect
- A majority would consider this a trivial effect
- Most would consider this a trivial effect
- All or almost all would consider this a trivial effect’

In the remaining scenarios, the survey in this hypothetical example would present scenarios in which the magnitude of the effect of laparoscopic cholecystectomy on reducing recurrent admissions at 90 days varied. When a panellist switches their response from ‘a majority would consider this an important effect’ to ‘a majority would consider this a trivial effect’ (or vice‐versa), the panellist will have identified a narrow range within which the minimal important difference lies [11]. We will review the minimally important difference thresholds derived from the online survey at the in‐person consensus meeting for approval from the panel.

### 2.10 Risk of Bias Appraisal

Both trainee reviewers will be calibrated to perform consistent appraisals of the risk of bias of eligible articles. We will use the ROBUST‐RCT tool for randomized controlled trials and the Tool to Assess Risk of Bias in Cohort Studies [12, 13]. The Systematic Review Lead will be available to resolve disagreements as they arise and will hold a synchronous meeting to resolve discrepancies if needed.

### 2.11 Methodology for Data Analysis

We will apply a random effects meta‐analysis to synthesize the evidence for our key question [14]. We will conduct meta‐analyses of proportions of events to calculate the baseline risk for each outcome, for the purposes of estimating absolute effect differences [15]. We will calculate Mantel–Haenszel pooled odds ratios for binary variables and the standardized mean difference for continuous variables, with corresponding 95% confidence intervals. We will consider inconsistency by using the *I* ^2^ statistic to explore the percentage of variability of effect estimates that is due to metagenetic rather than sampling error [16]. Additionally, we will further explore heterogeneity by evaluating the *Q*‐statistic and the 95% prediction intervals that show the plausible range of effect size values in a given study setting [16, 17, 18]. In the presence of substantial unexplained inconsistency in terms of relevance of studies to the population, intervention, comparator or outcomes evaluated in our key questions, we will consider not downgrading for inconsistency, because the random effects model provides a conservative interval estimate which typically prompts downgrading for imprecision, accounting to some degree for the variation of effect estimates of individual studies. The independent data analysis group will perform analyses using the R statistical package version 4.0.3 using the meta package [15, 19], without involvement of the steering group or panel members.

We will compute the fixed‐effect pooled estimate as a sensitivity analysis. If both fixed and random effects pooled estimates are similar, we will report the random effects results. If results differ, then we have small study effects and we will consider how much trust shall we place to smaller studies [20]. Where means and *p* values, or confidence intervals will be given, the standard error and the standard deviation will be obtained by using the formula suggested by the Cochrane Collaboration [21]. Time‐to‐event data, also known as survival data, arise when we are interested not only in the event of an outcome but also on the time elapsing before this event is observed. Participants for whom we did not observe the event (censored) but contributed some time will be part of the analysis. For trials including time‐to‐event outcomes, we will extract the hazard ratio and the standard error of the logarithm of the hazard ratio [22]. If instead of the standard error, the corresponding 95% confidence interval or *p* value is reported, we can use them to estimate the standard error [21]. These are standard outputs from a Cox proportional hazards model [23]. If these data are not given, we can approximately estimate the hazard ratio using statistics estimated from a long‐rank analysis or from Kaplan–Meier curves [21, 24]. If some studies give the hazard ratio but others give risk ratios (or the number of events and sample size in each group), we will combine them in a sensitivity analysis. We will explore for small‐study effects using funnel plots and statistical tests (e.g., Egger's test) [25].

### 2.12 GRADE Certainty of Evidence

We will consider the risk of bias across studies, imprecision, inconsistency, indirectness and publication bias to rate the certainty of evidence [26, 27]. We will construct GRADE evidence summary profiles in MAGICapp. To perform the certainty of evidence rating, we will use the confidence interval of effect estimates in the context of the minimal important difference thresholds, as well as the sample size of patients across comparisons to evaluate imprecision [27]. We will consider the indirectness of comparisons based on their correlation with the PICO elements according to those listed in our inclusion criteria including the population, intervention, comparison and outcomes (Table 1) [27]. For outcomes that are informed by both randomized and nonrandomized evidence, we will follow GRADE guidance considering the certainty of evidence of randomized and nonrandomized studies first separately, prior to an integrative fashion to guide the decision to include one or both types of studies in evidence synthesis [28]. When RCTs have high certainty, we will not consider nonrandomized studies [28]. When RCTs are deemed not to have high certainty, we will examine the certainty of evidence from nonrandomized studies [28]. Where the certainty of evidence is similar, we will aggregate data [28]. Where the certainty of evidence is superior in nonrandomized studies compared to randomized studies, we will take evidence from nonrandomized studies [28].

### 2.13 Evidence‐to‐Decision Framework

The steering group will generate GRADE summary of findings tables for the in‐person panel consensus meeting, supervised by S. A. and F. C. We will begin the panel consensus meeting with a detailed overview of the guideline development methodology, prior to presenting the evidence summaries. The first half of the meeting will be dedicated to addressing concerns surrounding the best available evidence. The second half of the meeting will be dedicated to the evidence‐to‐decision framework, where we will resolve disagreements by consensus [29]. We will use the following factors during the evidence‐to‐decision framework [30]:

- Benefits and harms of the intervention
- Certainty of the evidence
- Values and preferences of patients and healthcare providers
- Resources required
- Acceptability of the intervention
- Feasibility of implementing the intervention
- Equity

We will consider all outcomes within a fully contextualized for all important and critically important outcomes during the evidence‐to‐decision process [31]. During this process, our GRADE auditor will provide their expert guidance on our methodology but will not be permitted to make judgements about evidence‐to‐decision domains or final recommendations. During the meeting, panel members will reach consensus on the direction and strength of the recommendation(s) through discussion, with the opportunity to suggest revisions or additional recommendations in alignment with GRADE methodology [32]. We will exercise voting only if absolutely necessary and will consider consensus as agreement among > 80% of panel members, and any issues where consensus was not achieved will be documented in the final guideline report.

### 2.14 Publication and Dissemination

We will publish this protocol in the *Guidelines International Network (GIN)* Journal. This clinical practice guideline will be published in *Surgical Endoscopy and Other Interventional Techniques* as the official journal of EAES. We will present the recommendations at the EAES and SAGES annual meetings. The steering group will also arrange for broader dissemination via peer‐reviewed scientific meetings, social media, letters to the editor and other channels.

### 2.15 Target Users and Implications

This guideline applies to gastrointestinal surgeons, interventional radiologists and any other healthcare professionals involved in the management of acute cholecystitis. Most importantly, this guideline can support patients in informing their decision for or against management options, depending on their values and preferences.

We will develop a patient‐friendly version of the guideline to support future patients with acute cholecystitis in informed decision‐making about their health. The systematic review, meta‐analysis, and evidence‐to‐decision framework will further identify evidence gaps for future areas of research.

### 2.16 Feedback

The steering group will gather feedback on the guideline report from social media, the EAES website, letters to the editor and other feedback mechanisms. This feedback will be considered in future updates to the guideline.

### 2.17 Monitoring and Updates

We will search clinicaltrials.gov for ongoing studies on the topic and we will investigate the trend of publications over time. We will specify accordingly the period of validity for these guidelines.

### 2.18 Strengths and Limitations

We will develop this clinical practice guideline according to robust methodological standards outlined by GRADE. The involvement of an expert health sciences librarian will improve the comprehensiveness of our search to ensure that all relevant evidence is captured. We will develop patient‐centred recommendations, informed by the evidence and the perspectives of a diverse multidisciplinary panel.

Limitations will include general challenges such as the element of subjectivity present with interpreting evidence, which we will aim to mitigate by adhering to robust methodology. Furthermore, patient partners may not reflect the opinions of all patients, and this will be considered during the evidence‐to‐decision framework. Additionally, the full search syntax was not available, though we report the final search string in the appendix. Finally, this guideline will not be intended to dictate clinical practice, and clinicians should use this guideline as a guide while making patient‐tailored decisions.

## 3 Conclusion

This clinical practice guideline will provide rigorous, evidence‐informed recommendations to guide healthcare professionals and other key stakeholders in making patient‐centred decisions in the management of patients with acute cholecystitis.

## Author Contributions

Bright Huo, Salvador Morales Conde and Stavros A. Antoniou contributed to conceptualization of the project. Bright Huo and Dimitris Mavridis contributed to manuscript drafting and editing. Salvador Morales Conde, Carolina Soledad, Alexander A. Tzanis, Alexandros‐Georgios Asimakopoulos, Gordon Guyatt and Stavros A. Antoniou reviewed and edited the manuscript.

## Ethics Statement

All members of the Guideline Development Group will declare financial, personal or intellectual conflicts of interest. Conflicts will be managed according to the principles of the Guidelines International Network (GIN). Any member with relevant conflicts will participate as external advisors and will not participate in discussions about the direction or strength of the recommendations, the voting procedure, or the Delphi process following the consensus meeting.

## Conflicts of Interest

The authors declare no conflicts of interest.

## Supporting information


Supplementary Appendix 1: COI.

Supplementary Appendix 2: Search Syntax.

## Data Availability

All data are shared.
