# Supplementary material for: European Association of Endoscopic Surgery Clinical Practice Guideline Protocol: Evidence‐Informed Recommendations on the Management of Cholecystitis With ESAIC and ESR Participation
Source: Clin Public Health Guidel. 2025 Sep 1;2(4):e70043. doi: 10.1002/gin2.70043 (PMC13563383; doi:10.1002/gin2.70043)
Supplement: Supplementary file 1 [file GIN2-2-e70043-s001.docx]

Search syntax

## KQ1: Conservative vs surgery

- PubMed

((Cholecystitis, Acute[MeSH Major Topic]) OR (acute cholecystitis[Title/Abstract]) OR (cholecystitis[MeSH Major Topic])) AND ((laparoscopy[MeSH Major Topic]) OR (Surgical Procedures, Operative[MeSH Major Topic]) OR (cholecystectomy[Title/Abstract]) OR (surgery[Title/Abstract]) OR (laparoscopic cholecystectomy[Title/Abstract])) AND ((conservative[Title/Abstract]) OR (non-operative[Title/Abstract]) OR (Anti-Infective Agents[MeSH Major Topic]) OR (Anti-Bacterial Agents[MeSH Major Topic]))

- Embase

#1	exp cholecystitis/ or exp acute cholecystitis/	32227

#2	exp laparoscopic cholecystectomy/ or exp cholecystectomy/	65406

#3	surgery/	867866

#4	antiinfective agent/ or antibiotic agent/ or anti-microbial.mp.	640807

#5	2 or 3	923610

#6	1 and 4 and 5	1317

#7	limit 6 to (english language and yr="2010 -Current")	1041

- CENTRAL

#1	MeSH descriptor: [Cholecystitis, Acute] explode all trees	139

#2	acute cholecystitis	1082

#3	MeSH descriptor: [Cholecystitis] explode all trees	422

#4	#1 OR #2 OR #3	1246

#5	MeSH descriptor: [Laparoscopy] explode all trees	9458

#6	MeSH descriptor: [Surgical Procedures, Operative] explode all trees	177714

#7	cholecystectomy	7904

#8	surgery	338203

#9	laparoscopic cholecystectomy	6183

#10	#5 OR #6 OR #7 OR #8 OR #9	402881

#11	conservative	16131

#12	non-operative	1169

#13	MeSH descriptor: [Anti-Infective Agents] explode all trees	41404

#14	MeSH descriptor: [Anti-Infective Agents] explode all trees	41404

#15	#11 OR #12 OR #13 OR #14	58075

#16	#4 AND #10 AND #15 with Cochrane Library publication date Between Jan 2010 and Feb 2025	81

## KQ2: Cholecystostomy vs surgery

- PubMed

((cholecystitis, acute[MeSH Major Topic]) OR (acute cholecystitis[Title/Abstract]) OR (cholecystitis[MeSH Major Topic])) AND ((laparoscopy[MeSH Major Topic]) OR (surgical procedures, operative [MeSH Major Topic]) OR (cholecystectomy[Title/Abstract]) OR (surgery[Title/Abstract]) OR (laparoscopic cholecystectomy[Title/Abstract])) AND ((cholecystostomy[Title/Abstract]) OR (percutaneous cholecystostomy[Title/Abstract]) OR (percutaneous drainage[Title/Abstract]) OR (drainage[Title/Abstract]))

- CENTRAL

#1	MeSH descriptor: [Cholecystitis, Acute] explode all trees	139

#2	acute cholecystitis	1085

#3	MeSH descriptor: [Cholecystitis] explode all trees	424

#4	#1 OR #2 OR #3	1251

#5	MeSH descriptor: [Laparoscopy] explode all trees	9522

#6	MeSH descriptor: [Surgical Procedures, Operative] explode all trees	178596

#7	cholecystectomy	7971

#8	surgery	339791

#9	laparoscopic cholecystectomy	6239

#10	#5 OR #6 OR #7 OR #8 OR #9	404751

#11	percutaneous drainage	963

#12	drainage	13244

#13	percutaneous cholecystostomy	39

#14	cholecystostomy	60

#15	#11 OR #12 OR #13 OR #14	13285

#16	#4 AND #10 AND #15 with Cochrane Library publication date Between Jan 2010 and Feb 2025	144

- Embase

#1	MeSH descriptor: [Cholecystitis, Acute] explode all trees	139

#2	acute cholecystitis	1082

#3	MeSH descriptor: [Cholecystitis] explode all trees	422

#4	#1 OR #2 OR #3	1246

#5	MeSH descriptor: [Laparoscopy] explode all trees	9458

#6	MeSH descriptor: [Surgical Procedures, Operative] explode all trees	177714

#7	cholecystectomy	7904

#8	surgery	338203

#9	laparoscopic cholecystectomy	6183

#10	#5 OR #6 OR #7 OR #8 OR #9	402881

#11	percutaneous drainage	959

#12	drainage	13183

#13	percutaneous cholecystostomy	39

#14	cholecystostomy	60

#15	#11 OR #12 OR #13 OR #14	13224

#16	#4 AND #10 AND #15 with Cochrane Library publication date Between Jan 2010 and Feb 2025	144

## KQ3: Conservative vs surgery >72 hours

- PubMED Mar 14, 2025

| Search number | Query | Sort By | Filters | Search Details | Results | Time |
| --- | --- | --- | --- | --- | --- | --- |
| 35 | ((((72 hours[tw]) OR (delayed[tw])) AND ((((((cholecystitis[MeSH Terms]) OR (cholecystitis[tw])) OR (acute cholecystitis[MeSH Terms])) OR (acute cholecystitis[tw])) OR (grade II cholecystitis[tw])) OR (grade III cholecystitis[tw]))) AND ((((((((nonoperative management[tw]) OR (nonoperative management[MeSH Terms])) OR (antibiotics[MeSH Terms])) OR (antibiotics[tw])) OR (conservative management[MeSH Terms])) OR (conservative management[tw])) OR (conservative treatment[MeSH Terms])) OR (conservative treatment[tw]))) AND ((((((((surgery[MeSH Terms]) OR (surgery[tw])) OR (laparoscopic cholecystectomy[MeSH Terms])) OR (laparoscopic cholecystectomy[tw])) OR (surgical management[MeSH Terms])) OR (surgical management[tw])) OR (surgical intervention[MeSH Terms])) OR (surgical intervention[tw])) | | | ("72 hours"[Text Word] OR "delayed"[Text Word]) AND ("cholecystitis"[MeSH Terms] OR "cholecystitis"[Text Word] OR "cholecystitis, acute"[MeSH Terms] OR "acute cholecystitis"[Text Word] OR (("grade"[All Fields] OR "graded"[All Fields] OR "grades"[All Fields] OR "grading"[All Fields] OR "gradings"[All Fields]) AND "ii cholecystitis"[Text Word]) OR "grade iii cholecystitis"[Text Word]) AND ("nonoperative management"[Text Word] OR (("nonop"[All Fields] OR "nonoperative"[All Fields] OR "nonoperatively"[All Fields]) AND ("organization and administration"[MeSH Terms] OR "disease management"[MeSH Terms])) OR "anti bacterial agents"[MeSH Terms] OR "antibiotics"[Text Word] OR "conservative treatment"[MeSH Terms] OR "conservative management"[Text Word] OR "conservative treatment"[MeSH Terms] OR "conservative treatment"[Text Word]) AND ("surgical procedures, operative"[MeSH Terms] OR "general surgery"[MeSH Terms] OR "surgery"[Text Word] OR "cholecystectomy, laparoscopic"[MeSH Terms] OR "laparoscopic cholecystectomy"[Text Word] OR (("surgical procedures, operative"[MeSH Terms] OR ("surgical"[All Fields] AND "procedures"[All Fields] AND "operative"[All Fields]) OR "operative surgical procedures"[All Fields] OR "surgical"[All Fields] OR "surgically"[All Fields] OR "surgicals"[All Fields]) AND ("organization and administration"[MeSH Terms] OR "disease management"[MeSH Terms])) OR "surgical management"[Text Word] OR (("surgical procedures, operative"[MeSH Terms] OR ("surgical"[All Fields] AND "procedures"[All Fields] AND "operative"[All Fields]) OR "operative surgical procedures"[All Fields] OR "surgical"[All Fields] OR "surgically"[All Fields] OR "surgicals"[All Fields]) AND "methods"[MeSH Terms]) OR "surgical intervention"[Text Word]) | 130 | 14:22:02 |
| 34 | (((((((nonoperative management[tw]) OR (nonoperative management[MeSH Terms])) OR (antibiotics[MeSH Terms])) OR (antibiotics[tw])) OR (conservative management[MeSH Terms])) OR (conservative management[tw])) OR (conservative treatment[MeSH Terms])) OR (conservative treatment[tw]) | | | "nonoperative management"[Text Word] OR (("nonop"[All Fields] OR "nonoperative"[All Fields] OR "nonoperatively"[All Fields]) AND ("organization and administration"[MeSH Terms] OR "disease management"[MeSH Terms])) OR "anti bacterial agents"[MeSH Terms] OR "antibiotics"[Text Word] OR "conservative treatment"[MeSH Terms] OR "conservative management"[Text Word] OR "conservative treatment"[MeSH Terms] OR "conservative treatment"[Text Word] | 713,009 | 14:21:39 |
| 33 | nonoperative management[tw] | | | "nonoperative management"[Text Word] | 4,921 | 14:21:08 |
| 32 | nonoperative management[MeSH Terms] | | | ("nonop"[All Fields] OR "nonoperative"[All Fields] OR "nonoperatively"[All Fields]) AND ("organization and administration"[MeSH Terms] OR "disease management"[MeSH Terms]) | 2,809 | 14:21:04 |
| 31 | (((((((surgery[MeSH Terms]) OR (surgery[tw])) OR (laparoscopic cholecystectomy[MeSH Terms])) OR (laparoscopic cholecystectomy[tw])) OR (surgical management[MeSH Terms])) OR (surgical management[tw])) OR (surgical intervention[MeSH Terms])) OR (surgical intervention[tw]) | | | "surgical procedures, operative"[MeSH Terms] OR "general surgery"[MeSH Terms] OR "surgery"[Text Word] OR "cholecystectomy, laparoscopic"[MeSH Terms] OR "laparoscopic cholecystectomy"[Text Word] OR (("surgical procedures, operative"[MeSH Terms] OR ("surgical"[All Fields] AND "procedures"[All Fields] AND "operative"[All Fields]) OR "operative surgical procedures"[All Fields] OR "surgical"[All Fields] OR "surgically"[All Fields] OR "surgicals"[All Fields]) AND ("organization and administration"[MeSH Terms] OR "disease management"[MeSH Terms])) OR "surgical management"[Text Word] OR (("surgical procedures, operative"[MeSH Terms] OR ("surgical"[All Fields] AND "procedures"[All Fields] AND "operative"[All Fields]) OR "operative surgical procedures"[All Fields] OR "surgical"[All Fields] OR "surgically"[All Fields] OR "surgicals"[All Fields]) AND "methods"[MeSH Terms]) OR "surgical intervention"[Text Word] | 5,067,482 | 14:21:00 |
| 30 | (((((antibiotics[MeSH Terms]) OR (antibiotics[tw])) OR (conservative management[MeSH Terms])) OR (conservative management[tw])) OR (conservative treatment[MeSH Terms])) OR (conservative treatment[tw]) | | | "anti bacterial agents"[MeSH Terms] OR "antibiotics"[Text Word] OR "conservative treatment"[MeSH Terms] OR "conservative management"[Text Word] OR "conservative treatment"[MeSH Terms] OR "conservative treatment"[Text Word] | 707,028 | 14:20:19 |
| 29 | ((72 hours[tw]) OR (delayed[tw])) AND ((((((cholecystitis[MeSH Terms]) OR (cholecystitis[tw])) OR (acute cholecystitis[MeSH Terms])) OR (acute cholecystitis[tw])) OR (grade II cholecystitis[tw])) OR (grade III cholecystitis[tw])) | | | ("72 hours"[Text Word] OR "delayed"[Text Word]) AND ("cholecystitis"[MeSH Terms] OR "cholecystitis"[Text Word] OR "cholecystitis, acute"[MeSH Terms] OR "acute cholecystitis"[Text Word] OR (("grade"[All Fields] OR "graded"[All Fields] OR "grades"[All Fields] OR "grading"[All Fields] OR "gradings"[All Fields]) AND "ii cholecystitis"[Text Word]) OR "grade iii cholecystitis"[Text Word]) | 877 | 14:19:52 |
| 28 | (72 hours[tw]) OR (delayed[tw]) | | | "72 hours"[Text Word] OR "delayed"[Text Word] | 475,809 | 14:19:48 |
| 27 | (((((cholecystitis[MeSH Terms]) OR (cholecystitis[tw])) OR (acute cholecystitis[MeSH Terms])) OR (acute cholecystitis[tw])) OR (grade II cholecystitis[tw])) OR (grade III cholecystitis[tw]) | | | "cholecystitis"[MeSH Terms] OR "cholecystitis"[Text Word] OR "cholecystitis, acute"[MeSH Terms] OR "acute cholecystitis"[Text Word] OR (("grade"[All Fields] OR "graded"[All Fields] OR "grades"[All Fields] OR "grading"[All Fields] OR "gradings"[All Fields]) AND "ii cholecystitis"[Text Word]) OR "grade iii cholecystitis"[Text Word] | 23,122 | 14:19:41 |
| 26 | surgical intervention[tw] | |  | "surgical intervention"[Text Word] | 74,089 | 14:18:57 |
| 25 | surgical intervention[MeSH Terms] | | | ("surgical procedures, operative"[MeSH Terms] OR ("surgical"[All Fields] AND "procedures"[All Fields] AND "operative"[All Fields]) OR "operative surgical procedures"[All Fields] OR "surgical"[All Fields] OR "surgically"[All Fields] OR "surgicals"[All Fields]) AND "methods"[MeSH Terms] | 152,633 | 14:18:53 |
| 24 | surgical management[tw] | |  | "surgical management"[Text Word] | 57,995 | 14:18:50 |
| 23 | surgical management[MeSH Terms] | | | ("surgical procedures, operative"[MeSH Terms] OR ("surgical"[All Fields] AND "procedures"[All Fields] AND "operative"[All Fields]) OR "operative surgical procedures"[All Fields] OR "surgical"[All Fields] OR "surgically"[All Fields] OR "surgicals"[All Fields]) AND ("organization and administration"[MeSH Terms] OR "disease management"[MeSH Terms]) | 272,829 | 14:18:47 |
| 22 | laparoscopic cholecystectomy[tw] | | | "laparoscopic cholecystectomy"[Text Word] | 16,401 | 14:18:43 |
| 21 | laparoscopic cholecystectomy[MeSH Terms] | | | "cholecystectomy, laparoscopic"[MeSH Terms] | 14,465 | 14:18:38 |
| 20 | surgery[tw] |  |  | "surgery"[Text Word] | 3,272,632 | 14:18:34 |
| 19 | surgery[MeSH Terms] | |  | "surgical procedures, operative"[MeSH Terms] OR "general surgery"[MeSH Terms] | 3,739,969 | 14:18:32 |
| 18 | conservative treatment[tw] | | | "conservative treatment"[Text Word] | 42,676 | 14:18:26 |
| 17 | conservative treatment[MeSH Terms] | | | "conservative treatment"[MeSH Terms] | 5,762 | 14:18:19 |
| 16 | conservative management[tw] | | | "conservative management"[Text Word] | 21,188 | 14:18:15 |
| 15 | conservative management[MeSH Terms] | | | "conservative treatment"[MeSH Terms] | 5,762 | 14:18:12 |
| 14 | antibiotics[tw] | |  | "antibiotics"[Text Word] | 311,336 | 14:18:06 |
| 13 | antibiotics[MeSH Terms] | |  | "anti bacterial agents"[MeSH Terms] | 484,823 | 14:18:04 |
| 12 | delayed[tw] |  |  | "delayed"[Text Word] | 446,494 | 14:17:59 |
| 11 | 72 hours[tw] |  |  | "72 hours"[Text Word] | 30,982 | 14:17:55 |
| 10 | grade III cholecystitis[tw] | |  | "grade iii cholecystitis"[Text Word] | 6 | 14:17:26 |
| 9 | grade I cholecystitis - Schema: all | | | "grade"[All Fields] AND "cholecystitis"[All Fields] | 490 | 14:17:08 |
| 8 | Grade I cholecystitis[tw] | |  | grade i[Author] AND "cholecystitis"[Text Word] | 0 | 14:16:57 |
| 7 | Grade I cholecystitis[tw] - Schema: all | | | Grade I cholecystitis[tw] | 0 | 14:16:57 |
| 5 | grade II cholecystitis[tw] | |  | ("grade"[All Fields] OR "graded"[All Fields] OR "grades"[All Fields] OR "grading"[All Fields] OR "gradings"[All Fields]) AND "ii cholecystitis"[Text Word] | 5 | 14:16:45 |
| 4 | acute cholecystitis[tw] | |  | "acute cholecystitis"[Text Word] | 7,984 | 14:16:05 |
| 3 | acute cholecystitis[MeSH Terms] | | | "cholecystitis, acute"[MeSH Terms] | 3,050 | 14:15:54 |
| 2 | cholecystitis[tw] | |  | "cholecystitis"[Text Word] | 23,122 | 14:15:36 |
| 1 | cholecystitis[MeSH Terms] | | | "cholecystitis"[MeSH Terms] | 15,997 | 14:15:09 |

- Embase <1974 to 2025 March 13>

1	cholecystitis/	19507

2	cholecystitis.ti,ab,kf.	23614

3	acute cholecystitis/	11217

4	acute cholecystitis.ti,ab,kf.	10862

5	grade I cholecystitis.ti,ab,kf.	2

6	grade II cholecystitis.ti,ab,kf.	7

7	grade III cholecystitis.ti,ab,kf.	8

8	72 hours.ti,ab,kf.	60883

9	delayed.ti,ab,kf.	478673

10	antibiotics/	424399

11	antibiotics.ti,ab,kf.	368902

12	conservative management/	114589

13	conservative treatment/	114589

14	conservative management.ti,ab,kf.	30785

15	conservative treatment.ti,ab,kf.	51980

16	surgery/	877206

17	surgery.ti,ab,kf.	2206200

18	laparoscopic cholecystectomy/	12383

19	laparoscopic cholecystectomy.ti,ab,kf.	24033

20	surgical management/	877206

21	surgical management.ti,ab,kf.	76862

22	surgical intervention/	877206

23	surgical intervention.ti,ab,kf.	106844

24	1 or 2 or 3 or 4 or 5 or 6 or 7	34928

25	8 or 9	536824

26	24 and 25	1527

27	10 or 11 or 12 or 13 or 14 or 15	765534

28	16 or 17 or 18 or 19 or 20 or 21 or 22 or 23	2697642

29	26 and 27 and 28	249

30	nonoperative management/	18

31	nonoperative management.ti,ab,kf.	5852

32	10 or 11 or 12 or 13 or 14 or 15 or 30 or 31	769162

33	26 and 28 and 32	249

- COCHRANE

Search Name:

Date Run:	15/03/2025 02:42:39

Comment:

ID	Search	Hits

#1	cholecystitis:ti,ab,kw	2311

#2	MeSH descriptor: [Cholecystitis] explode all trees	421

#3	acute cholecystitis:ti,ab,kw	994

#4	MeSH descriptor: [Cholecystitis, Acute] explode all trees	138

#5	gradfe I cholecystitis:ti,ab,kw	0

#6	grade II cholecystitis:ti,ab,kw	122

#7	grade III cholecystitis:ti,ab,kw	49

#8	72 hours:ti,ab,kw	19843

#9	delayed:ti,ab,kw	38876

#10	antibiotics:ti,ab,kw	22068

#11	MeSH descriptor: [Anti-Bacterial Agents] explode all trees	16754

#12	MeSH descriptor: [Conservative Treatment] explode all trees	359

#13	conservative management:ti,ab,kw	4285

#14	conservative treatment:ti,ab,kw	12981

#15	surgery:ti,ab,kw	299897

#16	MeSH descriptor: [General Surgery] explode all trees	508

#17	laparoscopic cholecystectomy:ti,ab,kw	6056

#18	surgical management:ti,ab,kw	18990

#19	surgical intervention:ti,ab,kw	39619

#20	nonoperative management:ti,ab,kw	742

#21	#1 or #2 or #3 or #4 or #5 or #6 or #7	2311

#22	#8 or #9	57793

#23	#21 AND #22	173

#24	#10 or #11 or #12 or #13 or #14 or #20	46822

#25	#15 or #16 or #17 or #18 or #19	320193

#26	#23 and #24 and #25	49
