# Supplementary material for: European Association of Endoscopic Surgery Clinical Practice Guideline Protocol: Evidence‐Informed Recommendations on the Management of Cholecystitis With ESAIC and ESR Participation
Source: Clin Public Health Guidel. 2025 Sep 1;2(4):e70043. doi: 10.1002/gin2.70043 (PMC13563383; doi:10.1002/gin2.70043)
Supplement: Supplementary file 2 [file GIN2-2-e70043-s002.docx]

**Supplementary Appendix 2. Conflicts of Interest Form.**

1. Have you or your family received in the previous 12 months or anticipate receiving in the next 12 months income to participate in a study on an intervention that is subject to this guideline? This includes fees, travel, accommodation, meals, gifts, car lease, electronics, equipment, etc.
2. Have you or your family received in the previous 12 months or anticipate receiving in the next 12 months income to provide consultancy for a manufacturer of a technology or intervention relevant to this guideline? This includes fees, travel, accommodation, meals, gifts, car lease, electronics, equipment, etc.
3. Have you or your family received in the previous 12 months or anticipate receiving in the next 12 months payment for lectures and/or meeting attendance in support of a technology or intervention relevant to this guideline? This includes fees, travel, accommodation, meals, gifts, car lease, electronics, equipment, etc.
4. Have you or your family received in the previous 12 months or anticipate receiving in the next 12 months payment for board membership, patent applications and/or research grants (restricted or unrestricted) for a technology or intervention relevant to this guideline? This includes fees, travel, accommodation, meals, gifts, car lease, electronics, equipment, etc.
5. Have you published on a topic that expresses an opinion on the effectiveness of an intervention or currently performing research on a topic that could be affected by a recommendation of this guideline?
6. Do you anticipate receiving clinical income from a recommendation of this guideline?
7. Are you involved with an advocacy group that may gain from this guideline?
8. Do you have any political, religious, ideological, or other convictions or positions that may affect the subjectivity of your actions or decisions in the development of this guideline?
